# Supplementary material for: Next-Generation Sequencing of Connective Tissue Genes in Patients with Classical Ehlers-Danlos Syndrome
Source: Curr Issues Mol Biol. 2022 Mar 25;44(4):1472–8. doi: 10.3390/cimb44040099 (PMC9164033; doi:10.3390/cimb44040099)
Supplement: Supplementary file 1 [file cimb-44-00099-s001.zip › cimb-1623151-supplementary.pdf]

Table S1. Clinical findings of the 59 patients with cEDS (Supplementary Data).

Patient number	Gene	Variant	Protein	rs	Varsome	Major criteria			Minor criteria	Other features
						Skin hiper-extensibility	Atrophic scarring	Generalized joint hypermobility		
**1**	COL5A1	c.1989+1G>T	splice variant	not reported	Pathogenic	(+)	(+)	(+)	soft, velvety skin, easy bruising, dislocations of joints	poor wound healing, feet valgity, gastroesophageal reflux, astigmatism
**2**	COL5A1	c.1273_1276dupAGTC	p.Ser426Ter	not reported	Likely Pathogenic	(+)	(-)	(+)	soft skin, hernias, epicanthal fold, joint pain	delayed motor development, high palate
**3** (mother of patients 4 and 5)	COL5A1	c.5021delC	p.T1674Kfs*55	not reported	Pathogenic	(+)	(+)	(+)	soft, velvety skin, easy bruising, dislocations of joints, joint pain, family history of cEDS	poor wound healing, chronic fatigue, preterm delivery (patient 4 and 5), coronary arterial disease and chronic duodenal ulcer disease
**4** (daughter of 3)	COL5A1	c.5021delC	p.T1674Kfs*55	not reported	Pathogenic	(+)	(+)	(+)	soft, velvety skin, easy bruising, dislocations of joints, joint pain, family history of cEDS	poor wound healing, arrhythmia, chronic fatigue

**5** (daughter of 3)	COL5A1	c.5021delC	p.T1674Kfs*55	not reported	Pathogenic	(+)	(+)	(+)	soft, velvety skin, easy bruising, dislocations of joints, joint pain, family history of cEDS	poor wound healing, arrhythmia, mitral valve prolapse
**6** (mother of 7)	COL5A1	c.4050dupC	p.Gly1351Argfs*814	not reported	Likely Pathogenic	(+)	(-)	(+)	velvety skin, easy bruising, dislocations of joints, joint pain	flatfoot, preterm delivery of a daughter (patient 7) in 36 hbd and a son born in 27 hbd (not tested for cEDS)
**7** (daughter of 6)	COL5A1	c.4050dupC	p.Gly1351Argfs*814	not reported	Likely Pathogenic	(+)	(-)	(+)	soft, doughy skin, easy bruising, family history of cEDS	congenital hip dysplasia, scoliosis, mitral valve prolapse
**8**	COL5A1	c.1726C>T	p.Pro576Ser	rs763246328	Likely Benign	(+)	(+)	(+)	dislocations of joints, whole body pain	-
	COL6A3	c.6930+5G>A	splice variant	rs749037028	VUS					
**9** (described in [7])	COL5A1	c.944C>T	p.Thr315Met	rs145093766	Likely Benign	(+)	(-)	(+)	pain, family history of cEDS	flatfoot, scoliosis, chronic osteoporosis, short stature, muscle hypotonia, diarrhea, chronic fatigue syndrome
**10** (mother of patients 11-13)	COL5A1	c.3023C>T	p.Thr1008Met	rs199735010	Likely Benign	(+)	(-)	(+)	soft, velvety skin, dislocations of joints, family	varicose veins

									history of cEDS	
**11** (daughter of 10)	COL5A1	c.3023C>T	p.Thr1008Met	rs199735010	Likely Benign	(+)	(+)	(+)	soft, velvety skin, dislocations of joints, epicanthal fold, positive family history	scoliosis
**12** (daughter of 10)	COL5A1	c.3023C>T	p.Thr1008Met	rs199735010	Likely Benign	(+)	(+)	(+)	soft, velvety skin, dislocations of joints, joint pain, family history of cEDS	scoliosis, Raynaud's syndrome
**13** (daughter of 10)	COL5A1	c.3023C>T	p.Thr1008Met	rs199735010	Likely Benign	(+)	(+)	(+)	soft, velvety skin, dislocations of joints, whole body pain, family history of cEDS	
**14**	COL5A1	c.3398G>A	p.Arg1133Gln	rs759580799	Likely Benign	(+)	(+)	(+)	soft skin, dislocations of joints, joint pain	scoliosis, mitral valve prolapse
**15** (described in [7])	COL5A1	c.1089C>G	p.Asn363Lys	rs773870913	Likely Benign	(+)	(-)	(+)	easy bruising, dislocations of joints, flat feet,	-
**16** (described in [7])	COL5A1	c.193C>T	p.Arg65Trp	rs139468527	Benign	(+)	(-)	(+)	dislocations of joints, whole body pain	feet valgity, osteoporosis, high palate
	COL5A1	c.514G>T	p.Val172Phe	rs150147262	Likely Benign					
**17** (described in [7])	COL5A1	c.367C>G	p.Gln123Glu	rs142114921	Likely Benign	(+)	(-)	(+)	easy bruising, slow wound healing, joints dislocations	feet and knees valgity, high palate, chronic fatigue syndrome

**18** (described in [7])	COL5A1	c.4483G>A	p.Gly1495Ser	not reported	VUS	(+)	(-)	(+)	velvety skin, easy bruising, navel hernia, family history of cEDS	knees and heels valgity, scoliosis, muscle hypotonia, delayed motor development, high palate, reflux gastrointestinal, blue sclera
**19** (described in [7])	COL5A1	c.2588A>T	p.Glu863Val	rs139788610	Benign	(+)	(-)	(+)	velvety skin, easy bruising, dislocations of joints, family history of cEDS	congenital hip dysplasia, flatfoot and valgus foot, scoliosis, delayed motor development, osteoporosis, arrhythmia, mitral insufficiency, gastrointestinal disorders
	COL5A1	c.3418G>A	p.Val1140Met	rs149616140	Benign					
**20** (described in [7])	COL5A1	c.3418G>A	p.Val1140Met	rs149616140	Benign	(+)	(-)	(+)	velvety skin, dislocations of joints, family history of cEDS	-
**21**	COL5A2	c.2555G>A	p.Gly852Asp	not reported	Likely Pathogenic	(+)	(-)	(+)	soft, velvety skin, hernias, epicanthal fold	stretch marks, irritable bowel syndrome, bradycardia
**22**	COL1A1	c.2451T>C	p.Pro817=	rs374465457	Likely Pathogenic	(+)	(+)	(+)	soft, velvety skin, easy bruising, dislocations of joints, whole body pain,	-

									family history of cEDS	
**23**	COL1A1	c.517G>A	p.Gly173Arg	rs193922157	VUS	(+)	(+)	(+)	easy bruising, dislocations of joints, family history of cEDS	irritable bowel syndrome, mitral insufficiency, migraines, myopia, marfanoid habitus, ADHD (in childhood), suspicion of bleeding disorders
**24**	COL1A1	c.1984-5C>A	splice variant	rs66592376	Benign	(+)	(+)	(+)	soft, velvety skin, easy bruising with delayed wound healing dislocations of joints, joint pain	delayed motor development, high palate, pigeon chest (pectus carinatum), ADHD, a tendency to self-injury, hypersensitivity to sounds, MCAS (Mast Cell Activation Syndrome), mitral valve prolapse
**25**	COL1A2	c.601C>A	p.Pro201Thr	not reported	VUS	(+)	(+)	(+)	easy bruising, velvety skin, dislocations of joints, joint pain, and whole body pain	delayed wound healing, stretch marks, gastroesophageal reflux, irritable bowel disease, scoliosis, osteoporosis, osteomalacia,
	COL1A2	c.661G>A	p.Gly221Ser	not reported	VUS					

										chondromalacia, gluten intolerance
**26**	COL1A2	c.3706A>G	p.Ser1236Gly	rs781184808	VUS	(+)	(-)	(+)	soft, velvety, doughy skin,	collarbone fracture (1x), pigeon chest (pectus carinatum), blue sclera flatfoot, valgus knees
**27**	COL1A2	c.2776C>T	p.Arg926Cys	rs745363291	VUS	(+)	(+)	(+)	soft, velvety skin, easy bruising, dislocations of joints, joint pain	delayed wound healing, hand fracture in childhood (1x), hyperalgesia, pigeon chest (pectus carinatum), gothic palate, irritable bowel disease, POTS (Postural Tachycardia Syndrome), depressed mood
**28**	COL1A2	c.118C>A	p.Pro40Thr	rs1363689462	VUS	(+)	(+)	(+)	soft, velvety, doughy skin, easy bruising, delayed wound healing, dislocations of joints, joints and whole body pain	gastroesophageal reflux, irritable bowel syndrome
**29**	COL1A2	c.2642A>C	p.Glu881Ala	rs751201659	VUS	(+)	(-)	(+)	soft, velvety skin, easy bruising,	gastroesophageal reflux, hernia,

									delayed wound healing, hernias, dislocations of joints, joint pain	blue sclera, depressed mood
**30**	COL1A2	c.3313G>A	p.Gly1105Ser	rs139851311	VUS	(+)	(-)	(+)	soft, velvety skin, easy bruising	gothic palate
	COL6A3	c.4184G>A	p.Arg1395Gln	rs80272723	Benign					
	NOTCH1	c.3142C>T	p.Pro1048Ser	rs770521856	VUS					
**31**	no mutation	-	-	-	-	(+)	(+)	(+)	easy bruising, dislocations of joints	delayed wound healing, POTS (Postural Tachycardia Syndrome)
**32**	no mutation	-	-	-	-	(+)	(-)	(+)	soft, velvety skin, hernias, stretch mark, joint pain	irritable bowel syndrome
**33**	no mutation	-	-	-	-	(+)	(+)	(+)	soft, and velvety skin, dislocations of joints, joint pain	flatfoot, blue sclera
**34**	no mutation	-	-	-	-	(+)	(+)	(+)	soft, doughy skin, easy bruising, hernias, dislocations of joints, joints and whole body pain	delayed wound healing
**35**	no mutation	-	-	-	-	(+)	(+)	(+)	soft, velvety skin, dislocations of joints, joint pain	Raynaud's syndrome

**36**	no mutation	-	-	-	-	(+)	(-)	(+)	soft, velvety skin, dislocations of joints, joint pain	varicose veins, Raynaud's syndrome
**37**	no mutation	-	-	-	-	(+)	(+)	(+)	soft, velvety, easy bruising skin, dislocations of joints, joint pain	poor wound healing, mitral valve prolapse
**38**	no mutation	-	-	-	-	(+)	(+)	(+)	soft skin, hernias, dislocations of joints, joint pain	flatfoot, scoliosis
**39**	no mutation	-	-	-	-	(+)	(-)	(+)	soft, velvety skin, easy bruising with hernias, dislocations of joints, joint pain	delayed wound healing, flatfoot, gastroesophageal reflux, blue sclera
**40**	no mutation	-	-	-	-	(+)	(-)	(+)	soft skin, hernias, epicanthal fold, joint pain	delayed motor development, high palate, blue sclera
**41**	no mutation	-	-	-	-	(+)	(+)	(+)	dislocations of joints, whole body pain	POTS
**42**	no mutation	-	-	-	-	(+)	(+)	(+)	soft, velvety, doughy skin, dislocations of joints, pain of joints and whole body	delayed wound healing, gastroesophageal reflux, irritable bowel syndrome
**43**	no mutation	-	-	-	-	(+)	(-)	(+)	soft, and velvety skin,	stretch marks, irritable bowel

									hernias, joint pain	syndrome, blue sclera
**44**	no mutation	-	-	-	-	(+)	(+)	(+)	soft, doughy skin easy bruising, hernias, epicanthal fold, dislocations of joints, the pain of joints and whole body	gastroesophageal reflux, blue sclera
**45**	no mutation	-	-	-	-	(+)	(+)	(+)	dislocations of joints, joints and whole body pain, hernias	-
**46**	no mutation	-	-	-	-	(+)	(-)	(+)	soft, velvety skin, easy bruising with, hernias, dislocations of joints, joint pain	delayed wound healing, varicose veins, blue sclera
**47**	no mutation	-	-	-	-	(+)	(+)	(+)	soft, velvety skin, dislocations of joints, joint pain	delayed wound healing, gastroesophageal reflux
**48**	no mutation	-	-	-	-	(+)	(-)	(+)	soft, and velvety skin, hernias, epicanthal fold	stretch marks, irritable bowel syndrome, arrhythmia
**49**	no mutation	-	-	-	-	(+)	(-)	(+)	soft, velvety skin, hernias, joint pain	stretch marks, flatfoot, arrhythmia, irritable bowel

										syndrome, blue sclera
**50**	no mutation	-	-	-	-	(+)	(+)	(+)	soft, doughy skin, easy bruising, hernias, dislocations of joints, joint pain	gastroesophageal reflux
**51**	no mutation	-	-	-	-	(+)	(+)	(+)	soft, doughy skin, easy bruising, hernias, dislocations of joints	food intolerance
**52**	no mutation	-	-	-	-	(+)	(-)	(+)	soft, velvety skin, dislocations of joints, joint pain,	varicose veins, Raynaud's syndrome, blue sclera
**53**	no mutation	-	-	-	-	(+)	(+)	(+)	soft, velvety skin, easy bruising, dislocations of joints, whole body pain, family history of cEDS	-
**54**	no mutation	-	-	-	-	(+)	(-)	(+)	soft skin, hernias, epicanthal fold, joint pain	delayed motor development, high palate, blue sclera
**55**	no mutation	-	-	-	-	(+)	(+)	(+)	soft, velvety skin, dislocations of joints	delayed wound healing, gastroesophageal reflux

**56**	no mutation	-	-	-	-	(+)	(-)	(+)	soft, velvety skin, hernias, joint pain	delayed wound healing, stretch marks, irritable bowel syndrome, gastroesophageal reflux, blue sclera
**57**	no mutation	-	-	-	-	(+)	(-)	(+)	soft, velvety skin, easy bruising with delayed hernias, dislocations of joints, joint pain	wound healing, gastroesophageal reflux, blue sclera
**58**	no mutation	-	-	-	-	(+)	(-)	(+)	soft skin, hernias, epicanthal fold, joint pain	delayed motor development, high palate
**59**	no mutation	-	-	-	-	(+)	(+)	(+)	soft, velvety skin, easy bruising, dislocations of joints, whole body pain, family history of cEDS	-

Legend: (+) clinical feature detected, (-) clinical feature not detected, - do not concern
